# Development of on package indicator sensor for real-time monitoring of meat quality

**DOI:** 10.14202/vetworld.2015.393-397

**Published:** 2015-03-23

**Authors:** Vivek Shukla, G. Kandeepan, M. R. Vishnuraj

**Affiliations:** Division of Livestock Products Technology, Indian Veterinary Research Institute, Bareilly, Izatnagar, Uttar Pradesh, India

**Keywords:** buffalo meat, indicator sensor, meat quality, total volatile basic nitrogen

## Abstract

**Aim:**

The aim was to develop an indicator sensor for real-time monitoring of meat quality and to compare the response of indicator sensor with meat quality parameters at ambient temperature.

**Materials and Methods:**

Indicator sensor was prepared using bromophenol blue (1% w/v) as indicator solution and filter paper as indicator carrier. Indicator sensor was fabricated by coating indicator solution onto carrier by centrifugation. To observe the response of indicator sensor buffalo meat was packed in polystyrene foam trays covered with PVC film and indicator sensor was attached to the inner side of packaging film. The pattern of color change in indicator sensor was monitored and compared with meat quality parameters *viz*. total volatile basic nitrogen, D-glucose, standard plate count and tyrosine value to correlate ability of indicator sensor for its suitability to predict the meat quality and storage life.

**Results:**

The indicator sensor changed its color from yellow to blue starting from margins during the storage period of 24 h at ambient temperature and this correlated well with changes in meat quality parameters.

**Conclusions:**

The indicator sensor can be used for real-time monitoring of meat quality as the color of indicator sensor changed from yellow to blue starting from margins when meat deteriorates with advancement of the storage period. Thus by observing the color of indicator sensor quality of meat and shelf life can be predicted.

## Introduction

As the number of processes, products and packaging types increases, food companies are facing with the challenge of food safety concerns. As a society is becoming more complex, users (food producers, food processors, logistic operators, retailers and consumers) continuously demand innovative and creative food packaging to guarantee food safety, quality, and traceability. This requires appropriate technologies that can be integrated in food packaging. As a result, over the last decades, researches have shown a growing interest in developing different food safety measures to ensure security and quality of food [1]. The food industry is constantly trying to enhance the safety of products by acquiring new technologies [2]. To answer this, there is growing number of research in the field of intelligent packaging. According to European Union [3], intelligent packaging contains a component that enables the monitoring of the condition of packaged food or the environment surrounding the food during transport and storage. Intelligent packaging is thus a system that provides the user with reliable and correct information on the conditions of the food, the environment and/or the packaging integrity. It is an extension of the communication function of traditional food packaging and communicates information to the consumer based on its ability to sense, detect, or record changes in the product or its environment.

Intelligent packaging applications have been used for monitoring spoilage of meat and to predict its remaining shelf life. Indicator sensors have been developed for application in intelligent packaging using different indicator solution and carriers. Indicator sensors that are able to monitor volatile organic compounds and gas molecules such as H_2_, CO_2_, H_2_S, NH_3_, CH_4_ and total volatile basic nitrogen (TVBN) related to food spoilage are particularly required [4]. A chromogenic sensor array was prepared for monitoring boiled marinated turkey meat freshness using 13 indicators (including pH indicators, nucleophilic sensing dyes, etc.) and three different inorganic supports (i.e. UVM-7, alumina and silica gel [5]. A time temperature indicator containing chitosan/*polyvinyl alcohol* (PVA) films with anthocyanin from *Brassica oleraceae* (red cabbage) was prepared for application in intelligent food packaging to monitor the spoilage process [6]. A time-temperature indicator based on lactic acid was developed for monitoring food product quality [7]. A food spoilage indicator was developed for monitoring freshness of skinless chicken breast using two groups of pH sensitive dyes *viz*. Bromothymol blue and methyl red and bromothymol blue, phenol red and bromocresol green, which used CO_2_ as indicator metabolite for detection of spoilage [8]. A colorimetric gas sensor array based on natural pigments was developed for monitoring pork spoilage [9]. Mixture of bromothymol blue and phenol red were proposed for the fabrication of indicator sensor for monitoring chicken meat spoilage responsive to TVBN released from chicken meat during storage [10]. Curcumin ([1E, 6E]-1, 7-bis [4-hydroxy-3-methoxyphenyl] hepta-1,6-3dione) based indicator sensor which showed color response on reaction with TVBN was developed to monitor the production of TVBN and spoilage in shrimp [11].

Different indicator carriers were also used to carry indicator solution in food system. Agarose was used as carrier for myoglobin to detect hydrogen sulfide production during spoilage of unmarinated broiler cuts [12]. Different type of papers (untreated cellulose), polyamides, cellulose acetate, gels foam and resins were used as carriers for indicator solution with most preferred thickness of about 1 mm [13]. Polytetrafluoroethylene solid substrate was used to immobilize ammonia sensitive indicator dye, which showed changed spectral characteristics on exposure to volatile acidic and basic compounds [14]. An indicator carrier consisting of methylcellulose as binder and polyethylene glycol as plasticizers was developed to monitor spoilage of broiler meat [10]. Bacterial cellulose membrane made from *Acetobacter xylinum* culture was used as indicator carrier for curcumin [11].

Since the meat gets spoiled quickly on storage at ambient temperature, a packaging technology with an indicator sensor for monitoring spoilage would be really helpful in assuring the quality and safety of the packaged food product. Since, there is paucity of research on sensors; the present study was aimed to develop a new indicator sensor responsive to TVBN released from stored buffalo meat through a color response to predict degree of meat spoilage at ambient temperature.

## Materials and Methods

### Chemical used

Ethanol (Merck, Germany), filter paper (Merck, Germany), indicator solution (Merck, Germany) and other chemicals were procured from standard firms.

### Indicator solution

Different indicator solutions were analyzed either alone or in combination to check their efficiency of color response on reaction with volatile bases produced during storage of buffalo meat. Indicator solutions were selected on the basis of (1) Higher sensitivity to TVBN; (2) Higher dissociation constant (pKa): Measure of strength of acid/base and (3) Higher extinction coefficient: Ability to absorb light at a given wavelength.

Based on above characteristics bromophenol blue was selected as indicator solution of choice to be coated onto indicator carrier. Indicator solution (1% w/v) was prepared by dissolving bromophenol blue in 95% ethanol.

### Indicator carrier

Indicator carrier was selected on the basis of (1) ability to soak indicator solution; (2) ability to hold and prevent the leakage of indicator solution; (3) neutrality towards indicator solution; (4) Strength and durability while keeping along with meat under storage period. Based on above evaluated characteristics filter paper strip (Filter paper No.42) was selected as carrier for indicator solution.

### Indicator sensor

Indicator carrier was made into a standard size strip (1.5 cm × 5 cm) and centrifuged along with indicator solution for 15 min at 3000 rpm to coat indicator solution onto indicator carrier. Indicator carrier along with coated solution was dried at room temperature for overnight in dark to fabricate indicator sensor.

### Experimental setup

Fresh meat cuts (round portion) from buffalo bulls of the same age (1.5 years) were collected within 1 hr of slaughter from municipal slaughter house, Bareilly and placed immediately in the cooler box. Samples were immediately transported to the laboratory and carefully made into cuts of 500 g. Intact meat cut was packaged in sterilized convenient plastic tray (17 cm × 11 cm × 5.5 cm) covered tightly with cling film and indicator sensor was attached to inside of cling film keeping a distance of around 3 cm between meat and indicator sensor. Meat samples were kept at ambient temperature (25±1°C) and pattern of color change of indicator sensors upon reaction with TVBN released from stored buffalo meat in packaged head space was observed on each sample. Important meat quality parameters were analyzed in each buffalo meat samples after 6 and 24 h of storage to correlate color response of indicator sensor with different levels of spoilage so as to evaluate its applicability to monitor spoilage process in buffalo meat cuts.

### Monitoring physicochemical and microbiological changes in buffalo meat

Change in important physicochemical and microbiological parameters related to meat quality were recorded during storage to correlate gradual change in color of indicator sensor and deterioration in meat quality. TVBN content was estimated by micro diffusion technique described by Pearson (1968) [15]. Tyrosine value was estimated by the method of Strange *et al*. (1977) [16]. D-glucose content was estimated using a commercial kit (GAGO-1KT, Sigma Aldrich) as per the method described by Washko and Rice (1961) [17]. Total plate count was estimated according APHA (2001) [18].

### Statistical analysis

A total of 4 sample packages of the buffalo meat cut were taken at the appropriate time. All four samples were analyzed for TVBN, tyrosine value, D-glucose and total plate count; for increased accuracy, each sample determination was replicated three times (12 determinations in total per test condition). For purposes of statistical analysis, the average value of the three determinations was used per sample such that the statistics describe the variation between samples with n=4. The data generated for different quality characteristics were compiled and analyzed using SPSS (version 17.0 for windows; SPSS, Chicago, III., U.S.A.) with randomized block design and subjected to analysis of variance. Student’s t-test was carried out for comparing the difference between storage period. The smallest difference (D_5%_) for two means to be significantly different (p<0.05) was reported. The Pearson coefficient of correlation was also determined, and the significance (p<0.01) of correlation was reported.

## Results and Discussion

### Response of indicator sensor in stored meat

**Figure-1:**
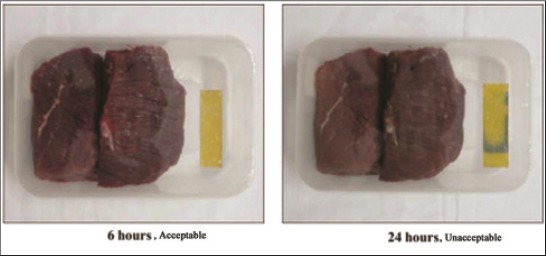
Color change of indicator sensor kept along with buffalo meat during storage. Yellow = Fresh, Bluish yellow = Unacceptable

Color of indicator sensor changed from yellow to blue starting from margins during the storage period (Figure-1). The color of indicator sensor was yellow at 6 h of storage, which changed to blue at 24 h of storage. The color change was not recorded further during the storage period as microbial count of buffalo meat crossed log_10_ 7 cfu/g of meat at 24 h of storage, which is the limit of rejection [19]. Color change in indicator sensor was due to the reaction of indicator sensor with volatile bases produced during storage of buffalo meat. Moreover, no visual difference was observed between sensors of the different sample batches. With the increase in storage period, there was an increase in production of volatile bases (Table-1), which on reaction with indicator sensor changed its color from yellow to blue starting from margins.

**Table-1:** Change in physicochemical and microbial parameters in buffalo meat at ambient storage.

Parameters			

Storage period (hours)	6	24	t value
Tyrosine value (mg/100 g)	21.81±0.14	44.30±0.16	196.498*
TVBN (mg/100 g)	8.45±0.4	19.15±1.32	8.26*
D-glucose (mg/100 g)	151.75±0.28	99.35±0.21	108.964*
Total plate count (log cfu/g)	4.44±0.23	7.80±0.45	217.452*

TVBN=Total volatile basic nitrogen, n=4,

* =p<0.05

### Comparison of color change of the indicator sensor with quality changes in buffalo meat during the storage period

**Figure-2:**
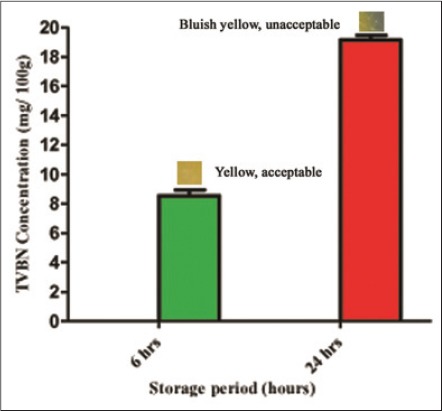
Change in color of indicator sensor with respect to change in concentration of total volatile basic nitrogen during ambient storage.

The concentration of volatile bases, TVBN increased significantly (p<0.05) during the storage period, which was evident by the color response of indicator sensor (Table-1). Although TVBN concentration did not cross the limit of 20 mg/100 g recommended for spoilage [20], microbial count reached beyond log_10_ 7 cfu/g. As a result meat was discarded, and study was conducted till 24 h of storage. Color response of the sensor was proportionate to the production of TVBN from stored meat (Figure-2). A marked correlation (r=0.914) was recorded between the microbial count of buffalo meat and the concentration of TVBN, which could be attributed to microbial growth, which produced TVBN. This correlation was in agreement with the findings of Rokka *et al*. (2004) [21] who observed a clear relationship between the microbiological quality of poultry (protein-based) and the total amount of biogenic amines.

**Figure-3:**
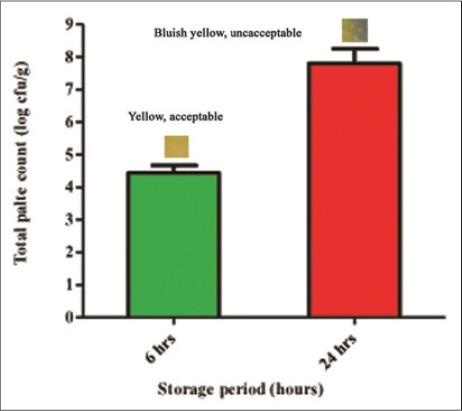
Change in color of indicator sensor with increase in total plate count in buffalo meat during ambient storage.

The total plate count of buffalo meat during storage increased significantly (p<0.05) with change in color of the indicator sensor from yellow to blue starting from margins during the same period (Figure-3). When compared to the sensor response it was observed that not only did the sensor response correlated with the changes in bacterial population but also that the sensor color changed from yellow to bluish yellow, which correlated with product rejection limit of 10^7^ cfu/g [19]. Moreover, the visual color changes of on-package sensors are useful indicators of the approximate microbial population and, therefore, the spoilage of the buffalo meat. Therefore, it is confirmed that indicator sensor can be used to confirm the presence of growing microbial population in aerobically packaged buffalo meat at ambient storage when color of indicator sensor changed to bluish yellow from yellow.

The tyrosine value of buffalo meat increased significantly (p<0.05) with gradual change in color of the indicator sensor from yellow to blue starting from margins at ambient storage (Table-1). Increased tyrosine value was due to hydrolytic changes in meat proteins produced by inherent tissue enzymes and bacterial proteases evident by a significant correlation (r=0.987) with total plate count during same period. In the present study increase in tyrosine concentration during storage was in compliance with color response of indicator sensor. Since, both tyrosine and TVBN are related to protein degradation; sensor response was closely related to increase in tyrosine value as for TVBN. A significant correlation (r=0.909) was also recorded between concentration of tyrosine and TVBN, which clearly indicated a strong association between them, thus with the color of indicator sensor.

**Figure-4:**
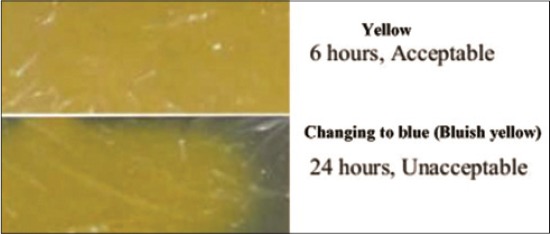
Color scale for indicator sensor designed to compare sensor response and freshness of buffalo meat at ambient temperature.

The D-glucose concentration decreased significantly (p<0.05) with gradual change in color of the indicator sensor from yellow to blue starting from margins during the storage period (Table-1). The result was attributed to use of glucose as the initial substrate for the growth and multiplication of microbes, after which nitrogenous compounds were used as source of energy [22]. The concurrent decrease in D-glucose concentration and increase in proteinaceous metabolite in buffalo meat and package head space changed the color of indicator sensor from yellow to bluish yellow during the storage period. With lowering of glucose concentration, microbial flora shifted to protein utilization manifested by significant (r=−0.910) negative correlation between TVBN and D-glucose. As a result, there was an increase in production of TVBN, which changed the color of indicator sensor from yellow to bluish yellow. Based on the above observations color scale was designed which can be attached to the package for comparing the color of indicator sensor at different stages of storage for monitoring meat quality (Figure-4).

## Conclusion

The strip type indicator sensor based on bromophenol blue coated onto the filter paper reacted with the volatile bases released from stored meat, which changed its color from yellow to blue starting from margins. This occurred upon exceeding the threshold value of TVBN for fresh meat, which was attained during the storage period of 24 h at ambient temperature. The relationship drawn between color change of indicator sensor and meat quality parameters indicated that the developed indicator sensor can be successfully utilized to predict degree of spoilage of buffalo meat just by observing the color of indicator sensor by naked eyes.

## Author’s Contribution

KG designed and supervised the research work. VS performed research work, data analysis and drafting of the manuscript. MRV helped in conducting the experiments. All authors read and approved the final manuscript.
